# Mechanical response of high-porosity rocks under high triaxial confining pressure and experimental study of indentation tests

**DOI:** 10.1371/journal.pone.0347234

**Published:** 2026-06-05

**Authors:** Jingming Gai, Wei Li

**Affiliations:** 1 Key Laboratory for Enhanced Oil & Gas Recovery of the Ministry of Education, Northeast Petroleum University, Daqing, China; 2 National Key Laboratory of Continental Shale Oil, Daqing, China; 3 National Engineering Research Center of Oil & Gas Drilling and Completion Technology, Daqing, China; Southwest Petroleum University, CHINA

## Abstract

As oil and gas exploration and development continue to advance, ultra-deep and extra-deep formations have become the primary battleground for increasing global oil and gas reserves and production. The influence of high formation pressure on the macro-mechanical response of rock, particularly the mechanical response of indentations, remains unclear. This paper takes high-porosity rocks, which are commonly found in ultra-deep and extra-deep formations, as the research object. True triaxial compression test(TTCT) and conventional triaxial compression tests(CTCT) were conducted on high-porosity red sandstone to analyze brittle-ductile transition characteristics. A high confining pressure indentation test apparatus was developed to investigate the mechanical response of spherical indentation under high confining pressure. Numerical simulations were employed to analyze the underlying mechanism of this mechanical response. The results indicate that as the confining pressure increases, the sample undergoes three failure modes: shear failure, dilatant failure, and compactive cataclastic flow. The peak points of the stress‒strain curves present two distinct failure surfaces on the p-q meridian plane, and the location of these failure surfaces on the p-q meridian plane largely determines the stiffness response of the indentation. The indentation stiffness does not always increase with the increase of confining pressure, but rather exhibits a significant reduction near the brittle-ductile transition point. The release of residual stress leads to significant side crack propagation during indenter unloading, but this phenomenon is significantly inhibited under confining pressure. This paper offers preliminary insights into the mechanical behavior of oil and gas drilling scenarios under high confining pressures, such as those in ultra-deep and extra-deep formations, and provides an initial understanding of the indentation mechanical response of high-porosity rocks under high confining pressure.

## Introduction

As oil and gas exploration and development continue to advance, ultra-deep and extra-deep formations have become the primary battleground for increasing global oil and gas reserves and production. The Gulf of Mexico has the largest number of ultra-deep and extra-deep wells in the world. According to statistics, more than 260 extra-deep wells with a completed drilling depth exceeding 9,000 meters have been drilled in this region [1]. With the continuous improvement of China’s drilling equipment and technology, the drilling of ultra-deep wells over 8000 meters has been normalized, and the drilling of 10-km deep wells has also achieved a breakthrough. As the well depth increases, drilling operations and oil production become more challenging, primarily due to the difficulties associated with high-pressure and high-temperature (HPHT) conditions [2]. Overpressure is generally developed in the basins of the Gulf of Mexico, especially in the central and northern marine areas of the basin which are ultra-high pressure zones, where the reservoir pressure can reach 210 MPa, and a large number of clastic hydrocarbon reservoirs with high porosity and high permeability are hosted [1,3]. Meanwhile, formation pressure in some regions of China has also reached a high pressure of 170 MPa [4]. According to American Petroleum Institute (API) standards API SPEC 6A, a well with a formation pressure greater than 138 MPa is defined as an Ultra-High Pressure (UHP) Well. It is noteworthy that although deep formations and geothermal wells exhibit high formation temperatures, this has a significant impact on the rheological properties of drilling mud and the temperature resistance of downhole tools, but only a minor effect on the mechanical properties of the rocks. Nearly all destructive chemical reactions of rocks occur at high temperatures exceeding 300°C to 400°C, while in most underground engineering projects, the rock temperature usually falls within a mild temperature range(<300°C) [5–7]. Therefore, in geological engineering, the influence of temperature on rock mechanical parameters is generally not taken into account in research, while pressure can significantly affect the mechanical properties of rocks [8–10].

At the end of the 20th century and the beginning of the 21st century, the failure mechanisms of rocks under such ultra-high pressure were studied by a small number of scholars, and their microscopic mechanisms were analyzed. However, this did not attract sufficient attention, with a relatively small number of studies conducted [11–17]. On the one hand, this research imposes extremely strict requirements on equipment; on the other hand, it is difficult to achieve such a level of confining pressure in practical engineering. However, with the substantive discoveries from field investigations [18,19] and the progress in drilling 10-km deep wells, the engineering value of this research has been significantly elevated. This is because locally compacted bands formed under ultra-high pressure may reduce local permeability, thereby creating a barrier to hydrocarbon flow [20].Under ultra-high pressure, localized plastic flow induced by pore collapse and shear-enhanced compaction serves as the main deformation mechanism for high-porosity rocks. However, as long as the pressure is sufficiently high, this mechanical behavior may also occur in low-porosity dense rocks [11].The variation in microscopic transport capacity resulting from localized rock deformation induced by ultra-high confining pressure is not the primary focus of this study. Instead, greater emphasis is placed on changes in macroscopic mechanical responses, particularly the brittle-ductile transition process under high confining pressure and its influence on the macroscopic indentation mechanical response of rocks.

Macroscopic indentation of rocks is the basic process of rock mechanical fragmentation, and conducting macroscopic indentation tests with a specific indenter is a core scientific method for evaluating this process [21]. The study of indentation mechanical behavior dates back to the 1880s, when Hertz [22] first investigated the cone cracking generated during the contact of glass lenses. Since then, indentation mechanics has been widely applied to the analysis and characterization of the fracture and deformation characteristics of ceramics, metals, and other materials [23], and has developed such advanced technical means as instrumented nanoindentation for evaluating the micromechanical parameters of materials [24]. The history of macroscopic indentation experiments can be traced back to the late 19th and early 20th century, a period when rock mechanics was emerging as a distinct field of study. These experiments were designed to analyze the fragmentation mechanisms of rocks and rock masses during indenter penetration, as well as to estimate the penetration forces on cutting tools and assess parameters such as rock brittleness and drillability [25–28].As rock mechanics is widely applied in fields such as mining, tunnel construction, and dam construction, research on rock indentation tests has surged, and experimental equipment and methods have been continuously improved [27,29–31].However, it is regrettable that among these experiments, those involving triaxial confining pressure were extremely scarce, and the applied pressures were notably low [32,33], because this kind of working condition is inconsistent with scenarios such as tunnel excavation and dam construction, it is thus not the focus of research [34–39]. However, triaxial confining pressure is of great importance to oil and gas drilling. Stratum rocks are subjected to triaxial confining pressure composed of geostress and the hydrostatic pressure of drilling fluid, and this pressure is extremely high, even reaching 200 MPa. Additionally, the brittle-ductile transition behavior of rocks under high confining pressure has not been taken into account in indentation tests. Thus, the mechanical response of rocks during indentation under triaxial high confining pressure remains a blank in current research.

This study analyzes the mechanical response, brittle-ductile transition process, and underlying mechanisms of high-porosity red sandstone samples under high confining pressure through true triaxial compression tests(TTCT) and conventional triaxial compression tests(CTCT). A high confining pressure indentation test apparatus was developed, and indentation tests with a spherical indenter were conducted to analyze the mechanical response of the indentation and the morphological characteristics of the crater. Finally, through numerical simulation, the study clarifies the stiffness response and fracture characteristics of spherical indenter indentation under high confining pressure, and discusses the influence of triaxial confining pressure on the indentation mechanical response.

## Triaxial compression test

### True triaxial compression test (TTCT)

**Fig 1:**
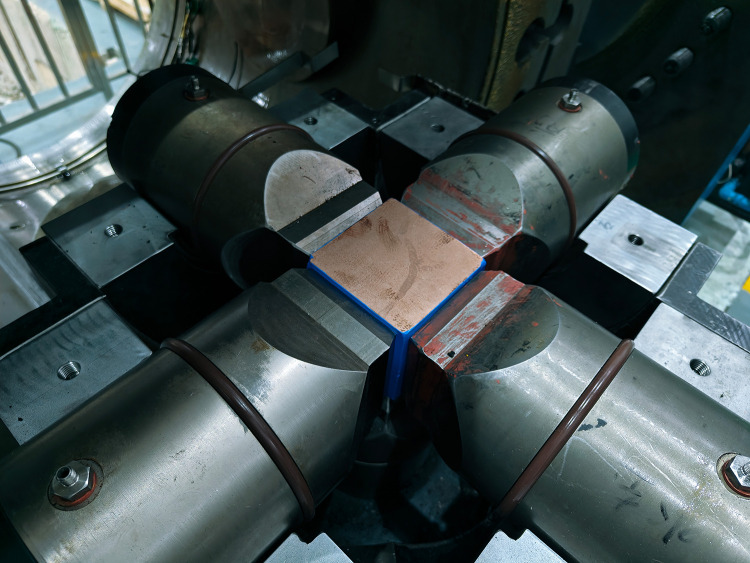
True triaxial compression test.

In this study, red sandstone was used as the research object, with a density of 2079 kg/m³ and a porosity of 15.05%. X-ray diffraction (XRD) results show that its mineral composition consists of quartz, plagioclase, calcite, and a small amount of clay. Thin section analysis indicates that the grain size of the rock ranges from 0.08 to 0.2 mm. This rock exhibits typical high-porosity characteristics, as its porosity is greater than the 5% cutoff porosity [11,40]. As shown in Fig 1, equal-pressure compression tests were conducted using the TAXW-5000 true triaxial testing system, which has a maximum load capacity of 5000 kN in each of its three principal directions. Cubic samples with dimensions of 100 mm × 100 mm × 100 mm were prepared. First, a preload of 50 kN was applied in three directions to fix the samples. Subsequently, displacement-controlled loading was performed at a rate of 0.1 mm/min until the load deviated from the hydrostatic pressure line.

**Fig 2:**
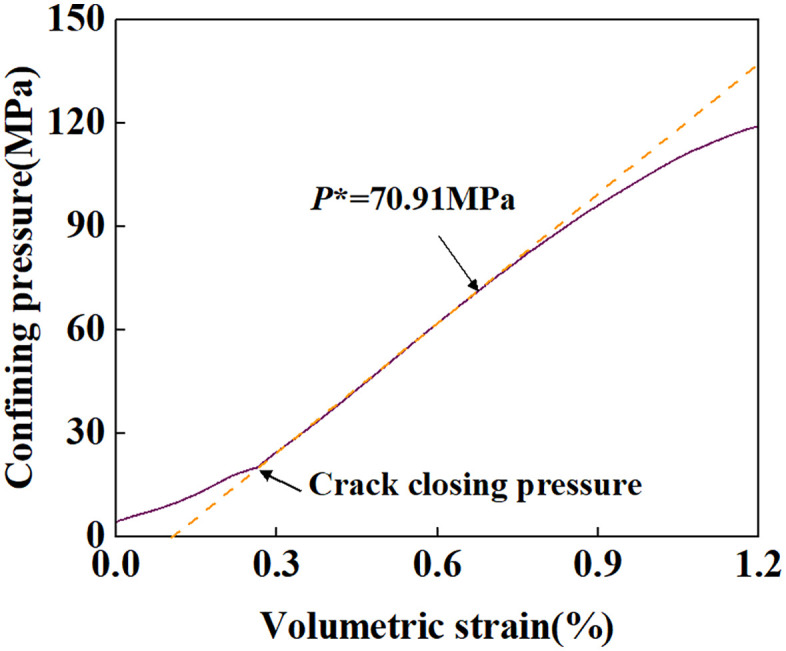
Confining pressure versus volumetric strain.

The relationship between confining pressure and volumetric strain is shown in Fig 2. Below 20 MPa, the sample was compressed until crack closure occurred. Subsequently, the pressure developed along the hydrostatic pressure line. At 70.91 MPa, the pressure deviated from the hydrostatic pressure line, and the sample exhibited significant inelastic strain. This point corresponds to the critical pressure for pore collapse $P^{*}$. As the pressure continued to increase to 120.48 MPa, the load became difficult to increase further, and the sample exhibited strain hardening. This point is recorded as the critical point for strain hardening $P^{*\prime }$. Unfortunately, due to the load capacity limitations of the testing machine, subsequent hardening curves could not be obtained in this study.

### Conventional triaxial compression test(CTCT)

#### Experimental procedure.

CTCT were conducted on the RTR-2000 electro-hydraulic servo rock triaxial testing machine, which has an axial load capacity of 2000 kN and a maximum confining pressure of 200 MPa. Based on the results of the TTCT, the confining pressures were set at 20, 40, 60, 80, 100, 130, 160, and 190 MPa. This allows the specimens to undergo pore closure, elastic deformation, pore collapse, and strain hardening during the confining pressure loading phase. Rock samples with a diameter of 25 mm and a height of 50 mm were prepared, and their end faces were ground and polished. It should be noted that although this size does not fully comply with the test standards of the International Society for Rock Mechanics (ISRM) and the American Society for Testing and Materials (ASTM), it is widely used in petroleum engineering. This is because obtaining intact large-sized samples from deep underground is extremely difficult, as high stress in the reservoir usually causes samples to crack during coring [41,42]. During sample installation, the sample was sheathed in a rubber membrane, connected to the base and top cap respectively, and clamped tightly. Subsequently, the pressure chamber was assembled.

First, the confining pressure was loaded to the predetermined value at a constant rate. After maintaining stability for a period of time, the axial load was applied. For experiments conducted below the critical pressure for pore collapse, the stress-controlled loading method was adopted. For experiments conducted above the critical pressure for pore collapse, due to the rock’s high ductility, the displacement-controlled loading method was employed, facilitating the acquisition of accurate post-peak stress-strain curves. For samples under confining pressure of 0 ~ 100 MPa, they were loaded to 4% axial strain; while those under confining pressure above 100 MPa were loaded to 8% axial strain, to observe the strain hardening after the stress reaches yield.

#### Stress-strain characteristics.

**Fig 3:**
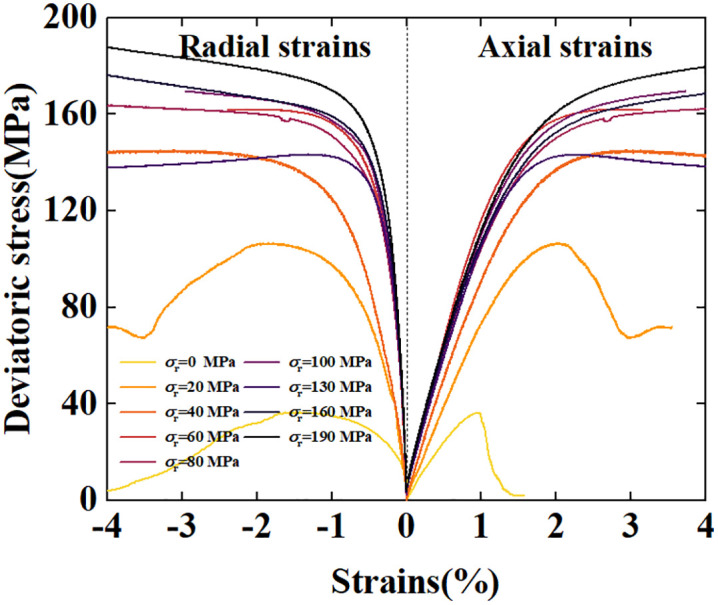
Deviatoric stress versus axial and radial strains.

The deviatoric stress- strain curve is shown in Fig 3. In this paper, strains are true and stresses are true. It can be observed that at low confining pressure levels, the peak stress increases significantly as the confining pressure rises. However, after the confining pressure increases to a certain level, the growth of the peak stress becomes slow. At confining pressures of 40 MPa and below, the stress-strain curve exhibits a marked post-peak drop, indicating brittle failure. At a confining pressure of 60 ~ 100 MPa, the stress-strain curve exhibits almost no post-peak drop characteristic; instead, it forms a strain-independent stress shelf. This indicates that as the confining pressure increases, the sample transitions from brittle failure to ductile failure. Combined with the results of the TTCT, it is considered that the failure mechanism of the sample at this stage is grain crushing and pore collapse.When the confining pressure exceeds 100 MPa, the stress shelf in the deviatoric stress-strain curve almost no longer exists; instead, it exhibits an upward trend. This is because during the confining pressure preloading stage, the pressure has already exceeded the critical pressure for pore collapse $P^{*}$ and even reached the critical pressure for strain hardening $P^{*\prime }$. As a result, grains and pores inside the rock have already collapsed, and the sample can only undergo minimal grain crushing or pore collapse under deviatoric stress, leading to strain hardening. This is consistent with the experimental results of Adamswiller sandstone and Rothbach sandstone under high confining pressures [43].

**Fig 4:**
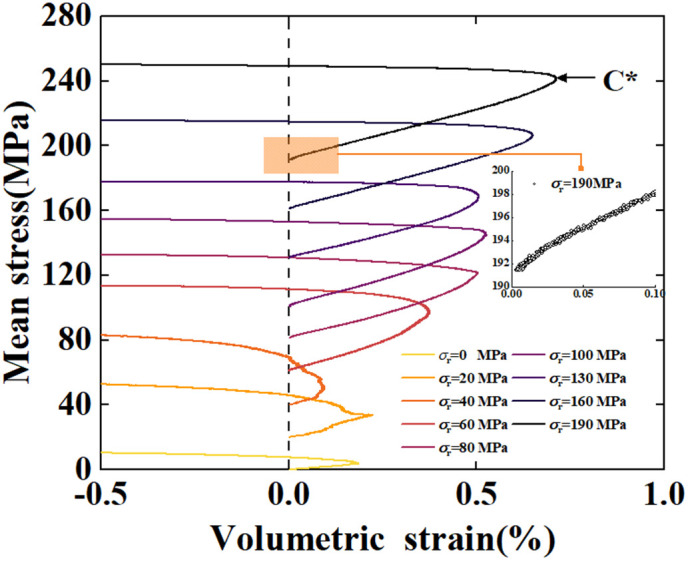
Mean stress versus volumetric strain.

The relationship between mean stress and volumetric strain is plotted as shown in the Fig 4. Since the TTCT results indicate that the mean stress does not always follow the hydrostatic pressure line, the volumetric strain in this figure is calculated starting from the deviatoric stress application stage, with the initial volumetric strain set to zero. The mean stress was increased to predetermined values during the confining pressure loading phase; therefore, the starting point of the mean stress under each confining pressure equals the applied confining pressure. In all groups, the volume of the samples decreased during the initial stage of loading (the volumetric strain increased). This indicates that the samples still underwent volume reduction in the early stage of deviatoric stress loading, manifesting sample compaction.With the increase of deviatoric stress, a rapid increase in volumetric strain deviating from the hydrostatic pressure line was observed during the initial deviatoric stress loading stage in groups with confining pressure > 80 MPa, as shown in the subfigures of Fig 4. This indicates that the rate of sample volume reduction accelerated, and deviatoric stress makes a significant contribution to the inelastic compaction of the sample, forming “shear-enhanced compaction”, but this process is very short. In addition, even if the confining pressure is higher than the critical pressure for strain hardening, shear-enhanced compaction can still occur, because this mechanical behavior is dominated by deviatoric stress [11–13,16,17]. With the continued loading of deviatoric stress, the sample was compressed to ultimate volume, exhibiting transient strain hardening (the mean stress increased rapidly), and then rapidly transitioned to dilatancy. The dilatancy inflection point is denoted as C*. The above analysis indicates that shear-enhanced compaction can be a transient precursor to dilatancy, and it is inappropriate to regard compaction and dilatancy as mutually exclusive processes. This is consistent with the research results of Baud [11].

**Fig 5:**
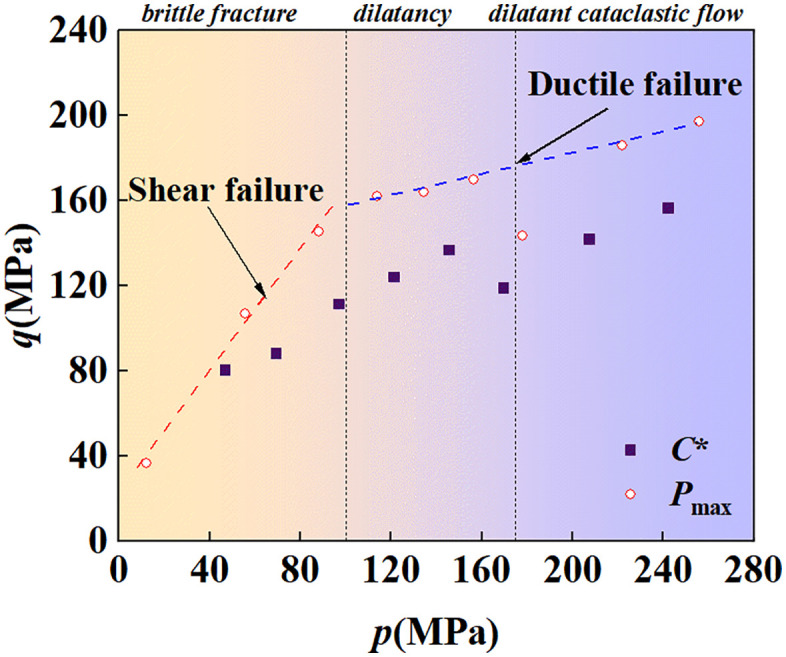
Failure surface in p-q meridian plane.

Plot the experimentally obtained peak deviatoric stress $P_{max}$ and dilatancy inflection point C* on the p-q meridian plane. As shown in Fig 5, the samples exhibited two distinctly different failure surfaces under low and high confining pressures. In terms of the macroscopic failure characteristics of the samples, shear failure occurred under low confining pressure, while ductile failure occurred under high confining pressure. The shear failure surface was characterized by a high internal friction angle and low cohesion, whereas the ductile failure surface was characterized by high cohesion and a low internal friction angle [44]. It should be noted that when the confining pressure exceeds the critical point of strain hardening $P^{*\prime }$, due to the strain hardening of the sample and the large strain (8% axial strain), the failure point on the ductile failure surface may lie slightly higher in the p-q space.

**Fig 6:**
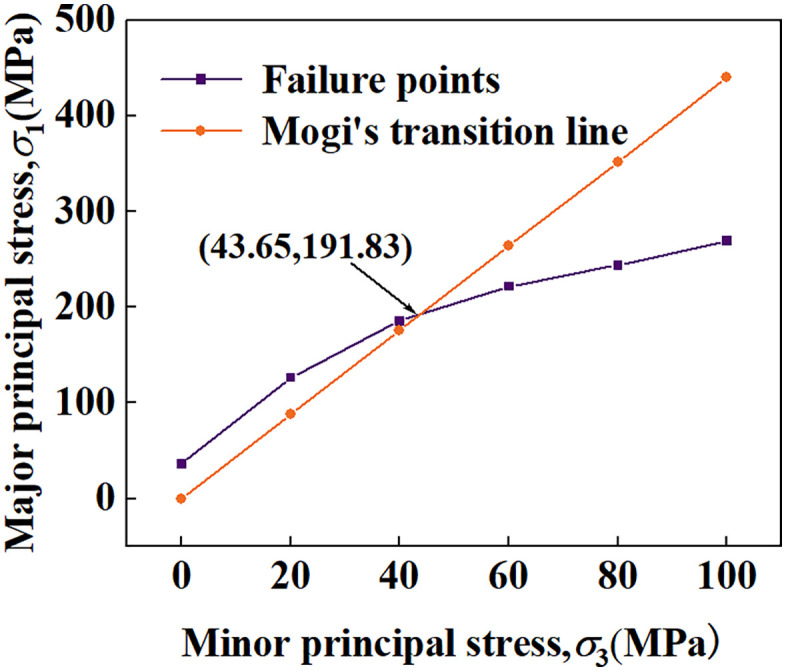
Brittle-ductile transition point using the Mogi method.

Plot the major and minor principal stresses under each confining pressure in Fig 6. The minor principal stress of the intersection point between the Mogi brittle-ductile transition line [45] ($\sigma_{\text{1}}=4.4\sigma_{\text{3}}$) and the $\sigma_{\text{1}}-\sigma_{\text{3}}$ line is 43.65 MPa, which indicates that the sample undergoes brittle-ductile transition at this point. This value is close to the uniaxial compressive strength. In terms of the macroscopic failure modes of the samples, there is almost no distinct boundary among their shear failure, dilatancy failure, and compactive cataclastic flow. Thus, a gradient color was used to represent the transition between these three failure modes.

#### Meso-macroscopic failure characteristics.

**Fig 7:**
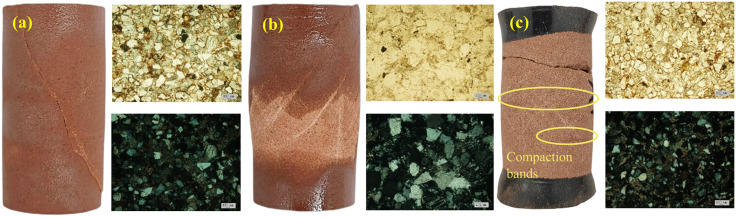
Failure modes of the samples under different confining pressures: (a) $\sigma_{r}=20$ MPa, single shear; (b) $\sigma_{r}=40$ MPa, double shear, dilatant bulging; (c) $\sigma_{r}=160$ MPa, compactive cataclastic flow, The sample exhibits a sandy texture with distinct compaction bands. The fracture shown in the image resulted from the sample becoming extremely brittle and powdery after testing, leading to damage during demolding.

With the increase in confining pressure, the failure modes of the rock undergo the typical processes of single shear, double shear, dilatant bulging, and compactive cataclastic flow as shown in Fig 7. The thin sections clearly reveal the microstructural characteristics of samples at different stages. During double-shear expansion failure, mineral grain size increases and particles undergo mutual compression, resulting in a significant reduction in pore volume. When forming a compactive cataclastic flow mineral grains are crushed into smaller particles and redistributed under pressure. It is noteworthy that in some experiments groups, visually observable radially distributed compaction bands were observed at the 1/3 distance from the sample end face, as shown in Fig 7c. Although the specimen exhibited volumetric expansion at 8% axial strain, the characteristic compaction bands formed by shear-enhanced compaction were retained.

Based on the characteristics of the stress-strain curve, yield surface features, and meso macroscopic failure characteristics of the specimens, this paper defines confining pressures ≤40 MPa as low-level confining pressures, while specimens exhibit brittle failure, with stress rapidly decreasing after reaching peak stress, resulting in macroscopic fracture. Defining confining pressures between 40 and 70 MPa as moderate levels, ductile failure occurs at this stage. The rock exhibits brittle-ductile transition behavior, displaying significant inelastic strain before reaching peak stress. Following the stress peak, a slight decrease in stress is observed. Confining pressures exceeding 70 MPa are defined as high-level confining pressures, at which point samples exhibit significant inelastic strain. Stress no longer decreases but instead reaches a shelf or undergoes strain hardening, leading to the formation of compactive cataclastic flow.

## Spherical indenter indentation experiment

### Experimental apparatus and procedure

**Fig 8:**
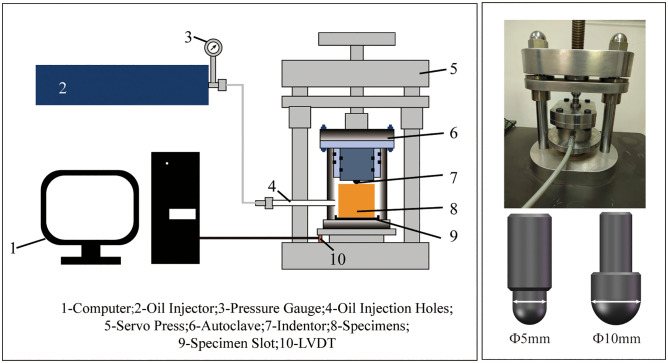
Indentation experimental apparatus.

Since Hertz first proposed the theory of elastic contact in 1881, the use of spherical or conical indenters to conduct quasi-static indentation tests for characterizing rock mechanical parameters and evaluating rock-breaking effectiveness has become widely adopted. Similar results are obtained for low-velocity impact problems [46–48]. However, due to the limitations of experimental conditions, research on the indentation mechanical behavior under confining pressure conditions still remains at the theoretical stage [49,50]. Very few experimental studies have been conducted, and the confining pressure levels employed are either relatively low [32,33] or hydrostatic pressure is not taken into account [34–39]. To investigate indentation mechanical behavior under triaxial high confining pressure, this study designed an indentation testing apparatus capable of withstanding 50 MPa pressure. This pressure slightly exceeds the brittle-ductile transition point, achieving a moderate level of confining pressure that permits the full development of ductile behavior in rock. As shown in Fig 8, the apparatus comprises: computer, oil injection pump, servo press, autoclave, spherical indenter, displacement and pressure sensors, and other components. The bottom of the autoclave features a ring-shaped groove that positions the rock at the center of the vessel, ensuring the indentation point aligns with the sample’s center. Two sets of sealing rings are installed inside the autoclave, located on the upper cover and the indenter column respectively. Since the indenter’s movement is quasi-static, it can withstand extremely high pressures. For safety reasons, the maximum experimental pressure is set at 50 MPa. During the experiment, plastic film was used to wrap the rock samples to prevent hydraulic oil from infiltrating the rock pores and affecting the pore pressure of the rock. After placing the sample into the autoclave, secure the upper cover with bolts. Inject hydraulic oil until the predetermined pressure is reached, maintain this pressure for 10 minutes, and then activate the servo press once the pressure has stabilized.

The indenters employed were Φ5 mm and Φ10 mm cemented carbide indenters. Due to the significantly higher hardness of the indenter material compared with the rock, the indenters can be regarded as rigid bodies.According to the recommendations of Yang [51] and Chen [52], the dimensions of rock specimens should be at least six times the plastic deformation caused by indentation. Setting the specimen dimensions to Φ100 mm × h50 mm serves two purposes: it avoids the Saint-Venant effect caused by excessively small dimensions, and facilitates the observation of specimen fracture at relatively low loading forces. This paper employs the cavity expansion model proposed by Alehossein [53] to calculate the size of the plastic zone under spherical indentation.


$$\begin{array}{c} \frac{d\xi_{*}}{d\delta }=\frac{1-\delta }{\delta (2-\delta )}[-\xi_{*}+\frac{\gamma \left(\delta \right)}{(1+\mu )\overset{2/K_{d}}{\underset{*}{\xi }}-\mu^{-2/K_{p}+1}}] \end{array}$$ (1)

where


$$\begin{array}{c} \xi_{*}=\gamma_{*}/a \end{array}$$ (2)

$$\begin{array}{c} \gamma \left(\delta \right)=\frac{\sqrt{\delta (2-\delta )}}{2\kappa (1-\delta )} \end{array}$$ (3)

$$\begin{array}{c} K_{p}=\frac{1+sin\left(\varphi \right)}{1-sin\left(\varphi \right)} \end{array}$$ (4)

$$\begin{array}{c} \kappa =\frac{(n+1)UCS}{2G(K_{p}+n)} \end{array}$$ (5)

Where $\xi_{*}$ is scaled radius of the elastic-plastic boundary. a is contact radius, $\gamma_{*}$ is radius of the elastic-plastic boundary. δ is scaled indentation depth, δ=h/R, h is actual penetration depth, R is radius of spherical indenter. μ is Poisson’s ratio, $K_{d}$ is dilatancy coefficient, $K_{p}$ is passive coefficient, n is dimension index (n = 1 for 2D and n = 2 for 3D problems). UCS is uniaxial compressive strength, G is shear modulus.

**Fig 9:**
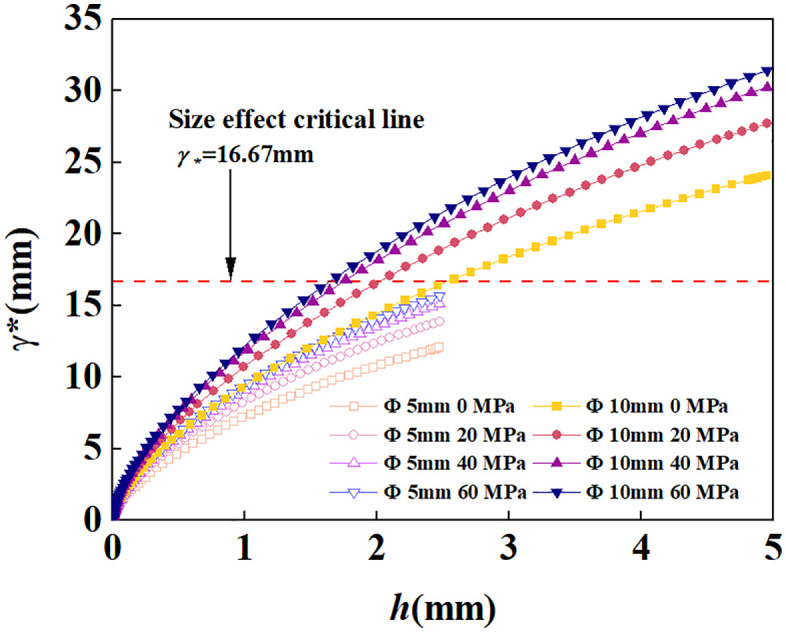
Relationship curve between indenter penetration depth and plastic zone radius.

The initial condition corresponds to $\xi_{*}=1$ at $\delta =\delta_{\text{0}}$, where $\delta_{\text{0}}$ is deduced from $\gamma \left(\delta_{\text{0}}\right)=1$. The differential equation in (1) was solved using the fourth-order Runge-Kutta method, and the $\gamma_{*}$ and h were determined as shown in Fig 9. The maximum plastic zone radius formed by a Φ5mm indenter consistently remains below the critical threshold for size effects, while a Φ10mm indenter exhibits size effects when penetration depth exceeds a certain value.

The servo machine applies a quasi-static load at a constant speed of 0.015 mm/s until the rock specimen fractures. The testing machine ceases loading upon detecting a sudden unloading of force (preset at 50 kg), enabling relatively accurate capture of the fracture force. Each indenter was tested under confining pressures of 0, 10, 20, 30, 40, and 50 MPa.

### Mechanical response of indentation

**Fig 10:**
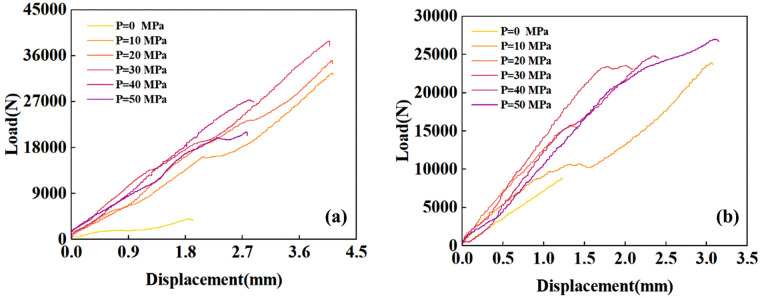
Load versus displacement curves: (a) Ф5mm indenter, (b) Ф10mm indenter.

The load-displacement curves obtained from the two-size indenter experiments are shown in Fig 10. During the experiments, all tests using the Φ10mm indenter identified fractures. However, tests with the Φ5mm indenter failed to accurately capture the sudden drop in force at confining pressures of 10, 20, and 30 MPa. Consequently, loading was halted at 4 mm, which represents the maximum penetration depth achievable with this indenter. After the experiment, the Φ10mm indenter group exhibited complete splitting, while the Φ5mm indenter group showed minor incomplete splitting at confining pressures of 20 and 30 MPa. This indicates that radial and side cracks in these groups propagated to the free surface, resulting in splitting. In terms of penetration depth, the Φ10 mm indenter group exceeded the critical threshold for triggering size effects at confining pressures of 10, 30, 40, and 50 MPa, whereas the Φ5 mm indenter group did not trigger size effects.

**Fig 11:**
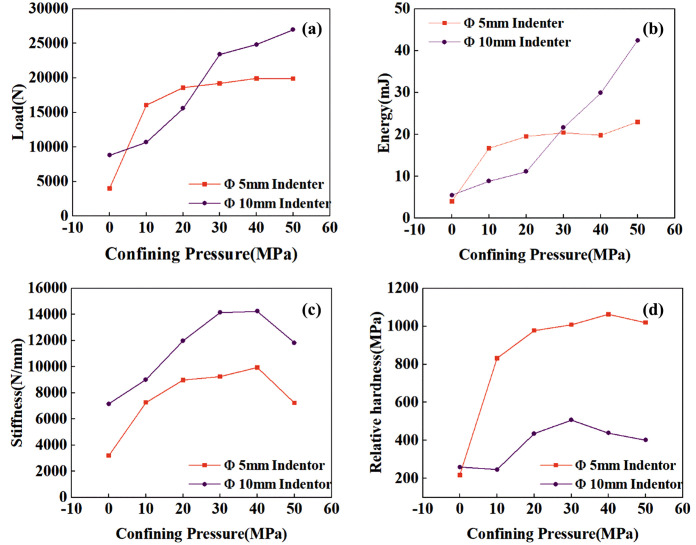
Load, energy, stiffness, relative hardness versus confining pressure.

Observation of the load-displacement curve and the crater morphology reveals that, although some curves did not exhibit a sudden drop in force, minor reductions in force were still present. This indicates that side cracks in the rock had fully developed, causing surface fragmentation. Following the force reduction, the force rose again and exhibited hardening behavior, demonstrating that the rock beneath the indenter underwent further compaction. Interestingly, for most groups subjected to confining pressure, the force did not exhibit a steep drop-off. This indicates that the specimens underwent significant plastic deformation rather than highly brittle failure, which differs markedly from the behavior observed under no confining pressure or lateral confining pressure alone [28,54]. The force, cumulative energy, stiffness, and relative hardness (ratio of indentation force to nominal projected area) at the point where the force first decreases are shown in Fig 11. For both the Φ10mm and Φ5mm indenter groups, the force and energy at the first fracture point increase with increasing confining pressure. Meanwhile, stiffness and relative hardness exhibit a trend of first increasing and then decreasing with confining pressure. This indicates that more energy is consumed in plastic deformation and crack propagation, and this transition relationship closely approaches the brittle-ductile transition point. This will be discussed in detail in subsequent analyses.

### Morphological characteristics of craters

**Fig 12:**
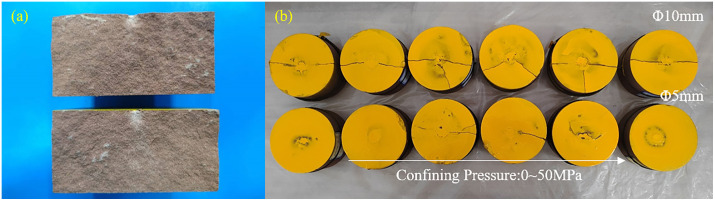
Cross-section of the sample and crater diagram.

**Fig 13:**
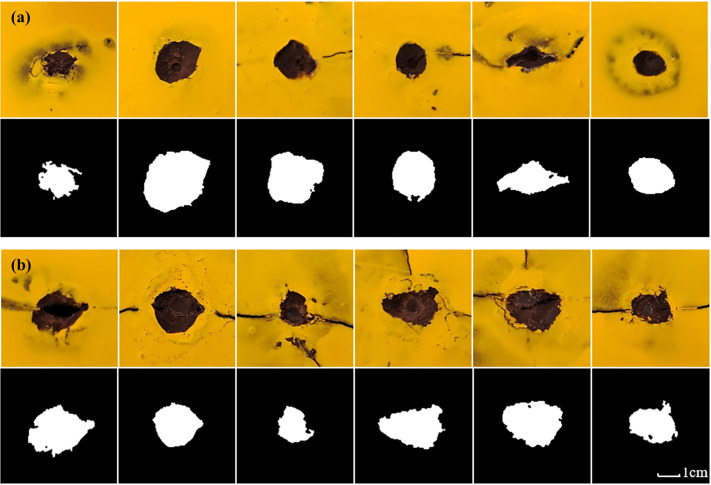
Morphology of craters formed by indentation under different confining pressures: (a) Ф5mm indenter, (b) Ф10mm indenter.

**Fig 14:**
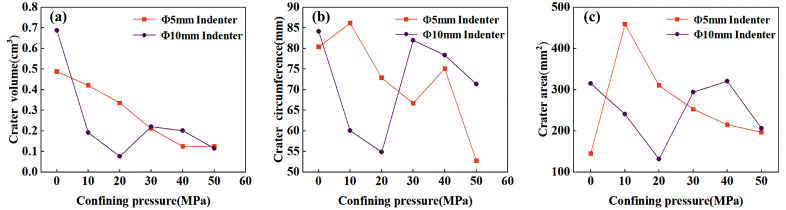
Relationship curve between crater volume, circumference, area and confining pressure.

The fractured surface of the rock exhibits distinct Hertzian failure characteristics, with a pronounced conical failure zone beneath the indenter, as shown in Fig 12a. To clearly observe the morphological features of the craters, a self-defoaming silicone with a hardness of 10 degrees was first configured. This silicone possesses excellent defoaming capabilities, along with favorable rheological properties and elasticity, ensuring thorough filling of cracks and enabling easy removal. Pour the prepared silicone into the crater until the surface is level, reserving 30 ml to test its density of 1.045 g/cm³. After the silicone mold has cured, spray the sample surface three times with yellow dye to ensure thorough coverage of the plastic regions along the raised crater edges. This prevents inaccurate edge detection during subsequent image recognition. The sprayed sample is shown in Fig 12b. After the silicone has fully cured, use tweezers to remove the silicone from the crater and weigh it to calculate the crater volume, as shown in Fig 14a. To enable quantitative analysis of the morphology of craters removed by the indenter, an image recognition program was developed. The photographed crater images were subjected to binarization and morphological processing, as shown in Fig 13, and the crater’s circumference and area were calculated, as depicted in Fig 14b and c.

Based on the failure modes of the samples and the morphology of the craters, two primary failure patterns resulting from indenter penetration were observed: First, complete splitting of the sample occurred in all experiments using the Φ10 mm indenter. Second, flaking of the free-surface rock surrounding the indenter primarily occurred during penetration with the Φ5 mm indenter. Despite inconsistent indentation displacement, fracture occurred only once in the sample, rendering the morphological analysis results reliable. As confining pressure increases, the volume, circumference, and area of the crater decrease in a power-law manner. The curve for the Φ5mm indenter is relatively smooth, while the curve for the Φ10mm indenter exhibits a jump at 30 MPa. Combining the load-displacement curve with the macroscopic splitting of the specimen and the dimensions of the plastic zone in the cavity expansion, this discontinuity is attributed to the combined effects of size effects and confining pressure. At lower confining pressures, the energy of the indenter penetrating the rock is primarily expended on splitting due to the minimal inhibition of crack propagation by confining pressure, resulting in smaller crater dimensions. As confining pressure increases, splitting is suppressed by confining pressure, and energy is increasingly directed toward plastic deformation, leading to a jump in the Φ10mm indenter group. Based on the size effect analysis results in Fig 9, the Φ5 mm indenter was unaffected by size effects and did not form through-type splitting, resulting in a continuous morphological curve for the crater. Interestingly, despite the decrease in crater volume and the area formed at the free surface as confining pressure increases, the value remains greater than the projected area of the indenter. This indicates that side cracks can always propagate to the free surface, yet their extension length is suppressed by confining pressure.

### Numerical simulation analysis of spherical indenter indentation under high confining pressure

In Fig 11. (c), the stiffness of the indentation load-displacement curve does not always increase linearly with confining pressure. Instead, a significant decrease occurs after the brittle-ductile transition pressure. This phenomenon was observed in both the Φ5 mm and Φ10 mm indenter experiments. Therefore, the author believes this is clearly not a coincidence. This pressure is very close to the brittle-ductile transition pressure, suggesting that the reduction in stiffness is likely related to the brittle-ductile transition. To verify this hypothesis, this study conducted numerical simulations of indentation by spherical indenters under high confining pressure.

### Modified Drucker-Prager cap model

The triaxial compression test results indicate that the high-porosity red sandstone selected for testing exhibits pore collapse and strain hardening characteristics under high confining pressure. This yield behavior at high confining pressure can be explained by the Drucker-Prager criterion with a cap [12,13,16,17]. Additionally, since Hertzian contact with spherical indenters can generate pressures as high as 10 GPa [55,56], this criterion is also applicable for describing the mechanical response of indentations [57].

The Modified Drucker-Prager Cap model (MDPC model) is widely applied in geotechnical engineering to describe the pressure-dependent yield behavior of materials under large-strain conditions. It demonstrates reliable predictive capability, particularly in characterizing compaction behavior [58]. This model is also employed to describe rock compaction under geological structural conditions [59].

**Fig 15:**
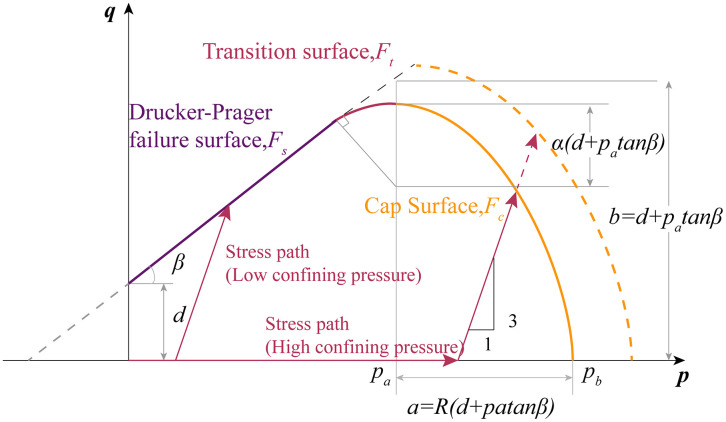
Modified Drucker-Prager cap model: yield surface in the p-q plane [60].

Fig 15 describes the yield surface state of the MDPC model in the p-q space, p is mean stress, q is Mises equivalent stress. For the cylindrical specimen of conventional triaxial test, $q=\sqrt{3J_{\text{2}}}=\left|\sigma_{z}-\sigma_{r}\right|$, $p=\frac{J_{\text{1}}}{\text{3}}=\frac{\sigma_{z}+2\sigma_{r}}{\text{3}}$, where $\sigma_{z}$ is axial stress, $\sigma_{r}$ is radial stress. The yield surface of the MDPC model comprises three primary components: the Drucker-Prager failure surface $F_{s}$ that provides shear flow, the transition surface $F_{t}$, and a cap surface $F_{c}$ intersecting the equivalent pressure axis.

The Drucker-Prager failure surface is written as:


$$\begin{array}{c} F_{s}=q-ptan\beta -d=0 \end{array}$$ (6)

where, tanβ is slope of the shear failure plane, β is internal friction angle, d is cohesion, the intercept of the q-axis.

The cap surface is dependent on the third invariant of stress. When yielding occurs on the cap surface, volumetric plastic compaction leads to hardening; whereas when yielding occurs on the shear failure surface, volumetric plastic dilation results in softening. The cap yield surface function is given by:


$$\begin{array}{c} F_{c}=\sqrt{(p-p_{a}{)}^{\text{2}}+[\frac{Rq}{\left(1+\alpha -\alpha /cos\beta \right)}{]}^{\text{2}}}-R(d+p_{a}tan\beta )=0 \end{array}$$ (7)

Where R is a material parameter that controls the shape of the cap, α is a small constant that determining the size of the transition surface, typically 0.01 to 0.05. $p_{a}$ an evolution parameter that represents the volumetric inelastic strain driven hardening/softening. The hardening/softening law is a user-defined piecewise linear function relating the hydrostatic compression yield stress $p_{b}$ and volumetric inelastic strain.


$$\begin{array}{c} p_{a}=\frac{p_{b}-Rd}{1+Rtan\beta } \end{array}$$ (8)

The MDPC model employs transition yield surfaces between the shear failure surface and the cap surface to eliminate singularity in the numerical model implementation, particularly when the stress state transitions from the cap surface to the failure surface. The yield surface equations used for the transition surfaces are as follows:


$$\begin{array}{c} F_{t}=\sqrt{(p-p_{a}{)}^{\text{2}}+[q-(1-\alpha /cos\beta )b{]}^{\text{2}}}-\alpha b=0 \end{array}$$ (9)

### Parameter calibration

Parameter calibration for the MDPC model is an extremely complex and challenging process, requiring a large number of laboratory experiments [61,62]. On the one hand, for rock materials, pore collapse typically requires extremely high confining pressures, imposing stringent demands on experimental conditions. On the other hand, the plastic volumetric strain data obtained from experiments often exhibit significant dispersion [63–65]. Given the difficulty of calibrating parameters via experimental means, numerical simulation optimization methods have emerged as a convenient approach for parameter calibration [58,66,67].

The initiation point of hardening, denoted as $p_{b0}=120.48$ MPa, was obtained from the TTCT, The fitting line of the failure surface was obtained from the CTCT. Parameters R and α, as well as the hardening curve, remain to be determined. It should be noted here that two distinctly different failure surfaces were observed in Fig 5. Specifically, under low confining pressures, the sample undergoes significant shear failure, which is inconsistent with the compaction behavior under large strains.

**Fig 16:**
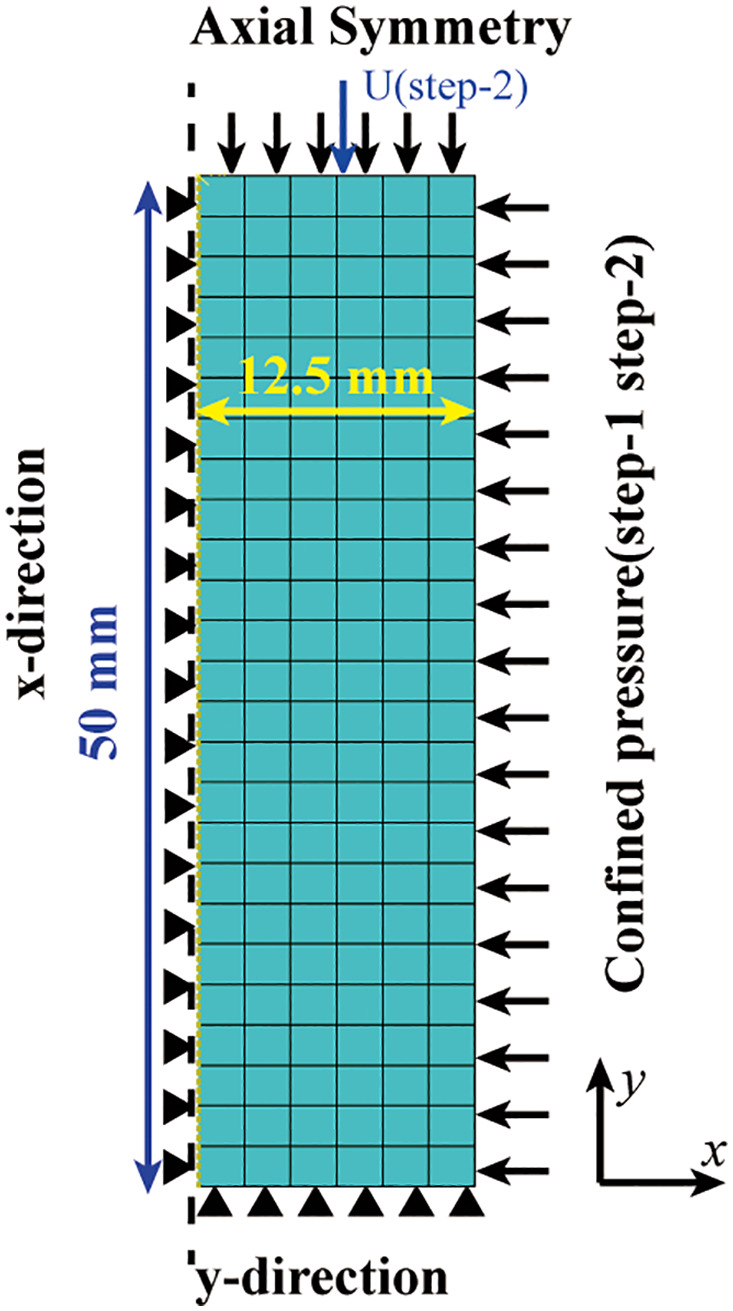
CTCT axisymmetric finite element model.

To determine the parameters of the constitutive criteria criteria, a axisymmetric model for CTCT was established, as shown in Fig 16. The sample has a width of 12.5 mm and a height of 50 mm. The model’s central axis and lower surface restrict displacement in the x and y directions, respectively. The mesh type is 4-node bilinear axisymmetric quadrilateral element, reduced integration CAX4R, element size is set to be uniform. Simulation performed according to ASTM D7012 using static analysis: In step 1, pressure is applied to the upper surface and side surfaces of the specimen. In step 2, a negative y-direction displacement is applied to the upper surface. The stress-strain behaviors under three confining pressures of 40 MPa, 80 MPa, and 160 MPa were simulated separately, corresponding to low, medium, and high confining pressures, respectively. In the MDPC constitutive criteria, the failure plane determines the height of the stress shelf, while R, α, and the hardening curve governs the shape of the stress-strain curve after yielding [58,68].

The parameters used are shown in Table 1. After repeated tests, it was found that if the shear failure surface in Fig 5 is adopted as the failure surface of the MDPC model, it will result in an excessively high post-yield stress shelf; whereas the use of the ductile failure surface as the failure surface yields results consistent with those of the CTCT. Therefore, this paper adopts the Drucker-Prager (DP) criterion for analysis under low confining pressures, whereas the MDPC model is employed for analysis under medium and high confining pressures.

**Table 1:** The parameter set of the DP model and MDPC model.

Confining pressure	Constitutive criteria	Parameters							
≤40 MPa	DP	Angle of friction(°)	Flow stress ratio	Dilation angle(°)		Yield stress(MPa)	Density (kg·m-3)	Young’s modulus (MPa)	Poisson’s ratio
		55	1	0		11.07	2750	7800	0.25
>40MPa	MDPC	β(°)	*d*(MPa)	*R*	*α*	Hardness law(MPa)			
		14	120	0.4	0.01	$p_{b}=120exp\left(20\overset{pl}{\underset{v}{\varepsilon }}\right)$			

**Fig 17:**
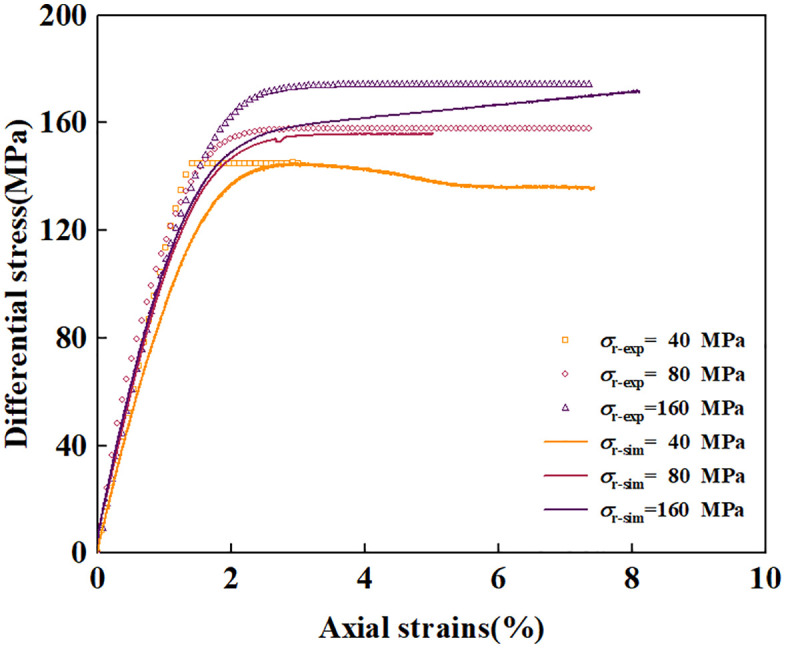
Comparison CTCT simulation results with experimental.

The comparison between numerical simulation results and experimental results is shown in Fig 17, where the stress-strain in the figure is nominal stress-strain. Under low confining pressure (40 MPa), the shear failure surface in Fig 5 governs the post-yield stress level of the sample, and the numerical simulation results are in excellent agreement with the experimental results. Since the shear failure criterion is not incorporated into the model, no post-peak stress drop is observed in the stress-strain curve. Under medium confining pressure (80 MPa), the stress path first reaches the hydrostatic pressure point in Step 1. Subsequently, in Step 2, as the axial displacement increases, the stress path evolves along a straight line with a slope of 3 on the p-q meridian plane until it intersects the cap surface. Strain hardening then occurs, and the stress path continues to evolve until it reaches the ductile failure surface in Fig 5. The height of the stress shelf is in excellent agreement with the experimental results. Under high confining pressure (160 MPa), the stress path already crosses the cap surface in Step 1. In Step 2, strain hardening occurs continuously until the stress path reaches the ductile failure surface. Since the position of the failure surface in this paper is determined by the deviatoric stress at 8% axial strain, the stress level at this strain condition is consistent with the experimental results.

### Indentation stiffness analysis

**Fig 18:**
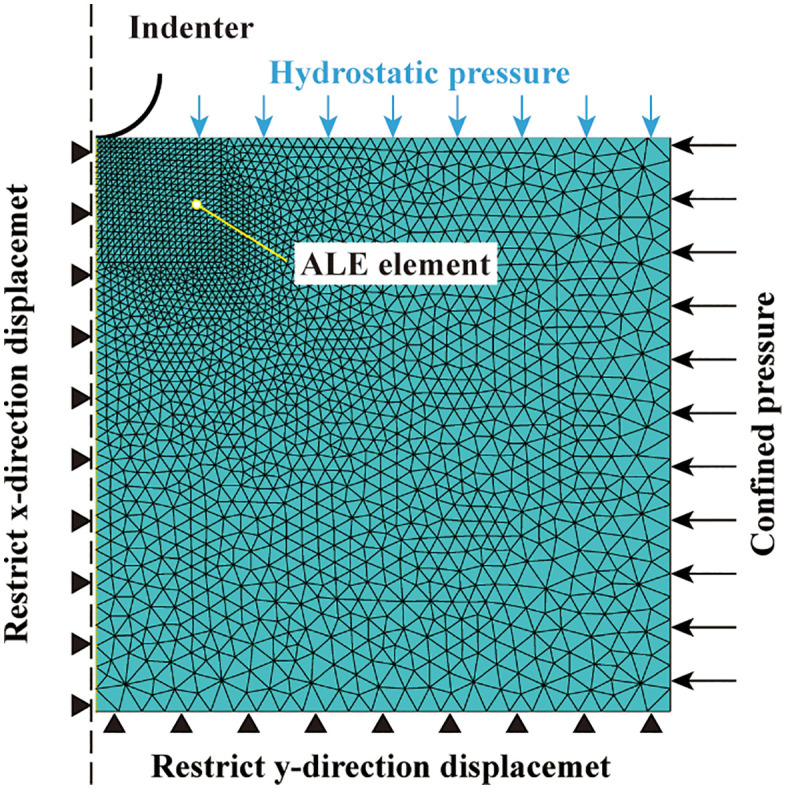
Axisymmetric model of spherical indenter penetration into rock.

The stiffness of the load-displacement curve provides crucial information, as it directly reflects the difficulty of the indenter penetrating the specimen. This paper establishes an axisymmetric model of a spherical indenter penetrating a specimen with dimensions of 50 mm × 50 mm, as shown in Fig 18. To avoid size effects, the indenter is modeled as a Φ5mm analytical rigid body. The mesh beneath the indenter is refined using an Eulerian-Lagrangian mesh to reduce data fluctuations during the initial indentation phase. The mesh adopts the 3-node linear axisymmetric triangular element CAX3, and the element size gradually increases from beneath the indenter toward the outer region, which not only ensures computational accuracy but also improves computational efficiency. The sample’s central axis is constrained from leftward displacement, and its lower boundary is constrained from downward displacement. In step 1, static analysis is employed, with hydrostatic pressure and confining pressure of equal magnitude applied to the upper and right boundaries of the sample, respectively, to simulate the pressurization process during the experiment. In Step 2, explicit dynamics analysis is employed, and the indenter is displaced downward by 3 mm. The reaction force and displacement of the indenter were recorded as functions of time. Since static analysis cannot be directly transferred to explicit dynamic analysis, a restart analysis was employed during this process.

**Fig 19:**
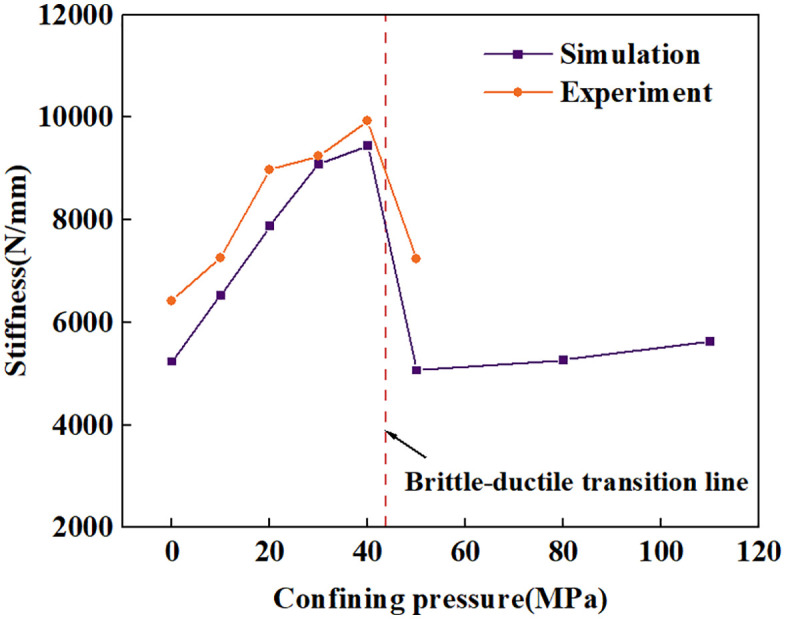
Stiffness-confining pressure relationship diagram for load-displacement curves.

As shown in Fig 19, the indentation stiffness obtained from numerical simulation is in very close agreement with the experimental results. Under low confining pressures, the stiffness increases almost linearly, and a significant drop in its value occurs at 50 MPa, which verifies the aforementioned hypothesis. When the confining pressure exceeds the brittle-ductile transition point, the indentation stiffness of the spherical indenter decreases, which is related to the transition of the failure surface. In addition, the indentation under higher confining pressures was also simulated, and the stiffness exhibited a slight increase.

**Fig 20:**
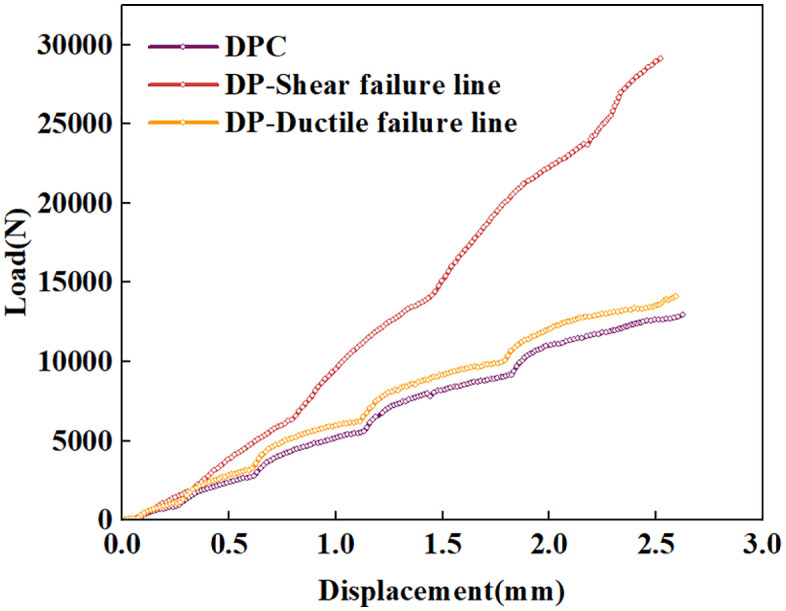
Indentation load-displacement curves of the three constitutive criteria under 80 MPa confining pressure.

**Fig 21:**
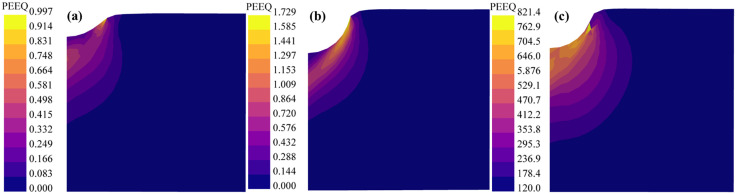
Equivalent plastic strain under the three constitutive criteria:(a)DP criteria with shear failure surface,(b) DP criteria with ductile surface,(c)MDPC criteria.

Under a confining pressure of 80 MPa, the indentation load-displacement curves and stress-strain distributions were compared for three cases: the Drucker-Prager (DP) criterion with the shear failure surface, the DP criterion with the ductile failure surface, and the MDPC model, as shown in Figs 20 and 21. When applying the DP criterion with shear failure surfaces, the stiffness of the load-displacement curve is significantly higher than in the other two cases, resulting in excessive discrepancies with experimental predictions. When employing the DP criterion with ductile failure surfaces, the stiffness of the load-displacement curve is slightly higher than that of the MDPC criterion. From the equivalent plastic strain contours, at an indentation depth of 3 mm, the equivalent plastic strain beneath the indenter is only 1.729 under the DP criterion, while that under the MDPC criterion is as high as 716.1. It is evident that the equivalent plastic strain under the DP criterion is excessively low, which is inconsistent with the actual conditions beneath the indenter. Therefore, under high confining pressures, the force response without the cap surface is higher, because the DP criterion cannot account for the highly constrained compressive stress state [57]. In addition, the two results obtained from the DP criterion with the shear failure surface and that with the ductile failure surface indicate that the spatial position of the failure surface on the p-q meridian plane governs the indentation stiffness response to a great extent.

### Fracture characteristic analysis

In the previous section, since no corresponding damage criteria were incorporated into the simulation, the failure mode of the spherical indenter penetrating the rock was not discussed. In this section, cohesive elements are incorporated into the model to simulate fracture behavior during indenter penetration. A program was developed to record the number of cohesive element failures over time, thereby calculating the evolution of crack volume and energy. A plane strain model was established to facilitate clearer observation of crack initiation and propagation direction during the indentation process, with boundary conditions identical to those under axisymmetric conditions. The loading process involves the indenter descending 2 mm within 10 seconds, followed by a 1-second unloading process where the indenter returns to its initial position. This loading rate does not affect the calculation results, as it is significantly lower than the wave velocity of the rock [47,69]. The cohesive element material parameters used are shown in Table 2. The normal failure stress is obtained from the Brazilian split test, while the tangential failure stress is derived from the cohesion during pure shear failure. Due to the extremely low unconfined compressive strength of the samples, the experimentally measured intrinsic fracture energies exhibited significant dispersion. Furthermore, since this section does not focus on the load at the failure point, the fracture energy is set to the commonly used intrinsic fracture energy for rocks [70]. According to Byerlee’s friction law, the sliding coefficient between rock elements is independent of rock type. When effective stress is less than 200 MPa, the friction coefficient is 0.85 [71,72]. In the field output, MMIXDME is used to represent the proportion of mixed fracture modes during damage evolution. A value of −1 indicates that the element has not failed. Values between 0 and 0.5 indicate that the element primarily fails by tensile failure, while values between 0.5 and 1 indicate that the element primarily fails by shear failure.

**Table 2:** Cohesive element material parameters.

Parameters	Elastic modulus(MPa)	Nominal stress of normal-only mode(MPa)	Nominal stress in the first direction(MPa)	Nominal stress in the second direction(MPa)	Fracture energy(N/mm)
Values	6e4	8.25	11.07	11.07	0.1

**Fig 22:**
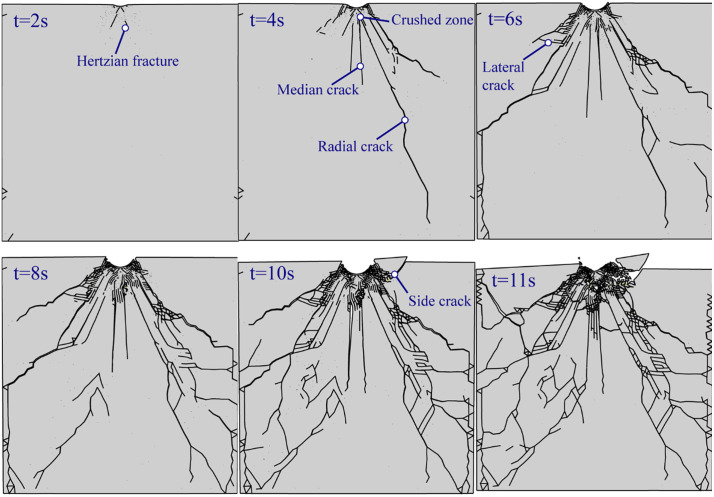
Fracture development under unconfined pressure over time.

**Fig 23:**
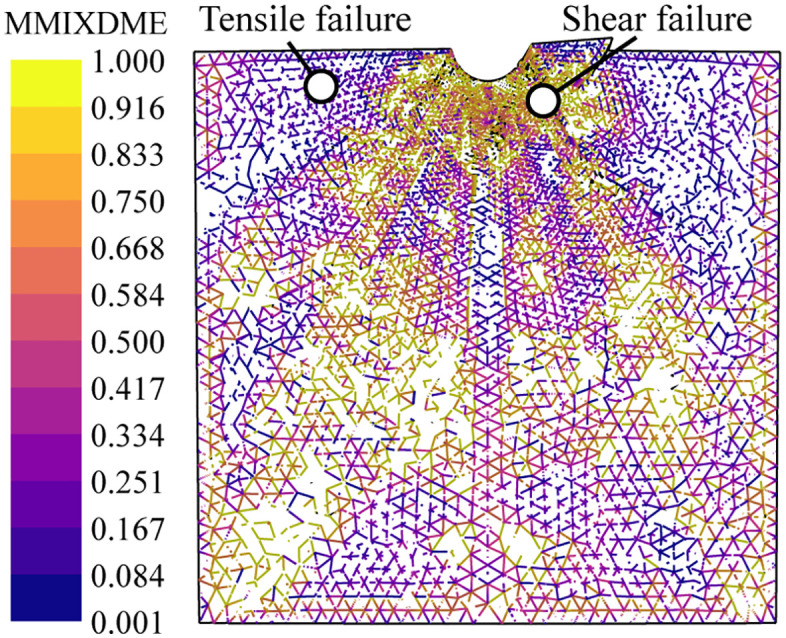
Damage mode distribution diagram of MMIXDME under unconfined pressure.

Fig 22 shows the crack propagation process of an indentation under unconfining pressure. The material beneath the button undergoes compaction and failure first, forming a fracture zone. Hertzian cone cracks and crushing zones develop initially. As the penetration force increases, radial cracks rapidly propagate following Hertzian cracks, while median cracks simultaneously initiate and develop. With the load continued to increase, causing lateral cracks to form near the radial cracks. However, these lateral cracks did not develop as rapidly as the radial cracks and the median cracks. Side cracks propagate to the free surface and further expand during the unloading phase. The MMIXDME diagram (Fig 23) shows that the median crack, lateral crack, and side crack exhibit significant shear failure, while the radial crack initially develops as shear failure and later transitions to tensile failure.

**Fig 24:**
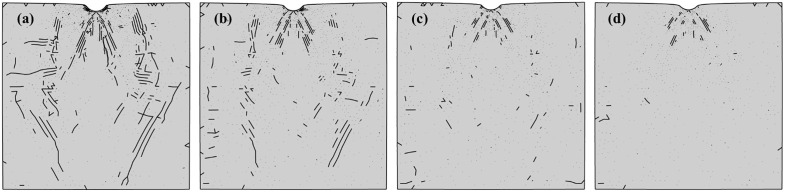
Crack distribution diagrams at the end of loading under different confining pressures, from left to right: (a) 20 MPa, (b) 40 MPa, (c) 60 MPa, (d) 80 MPa.

**Fig 25:**
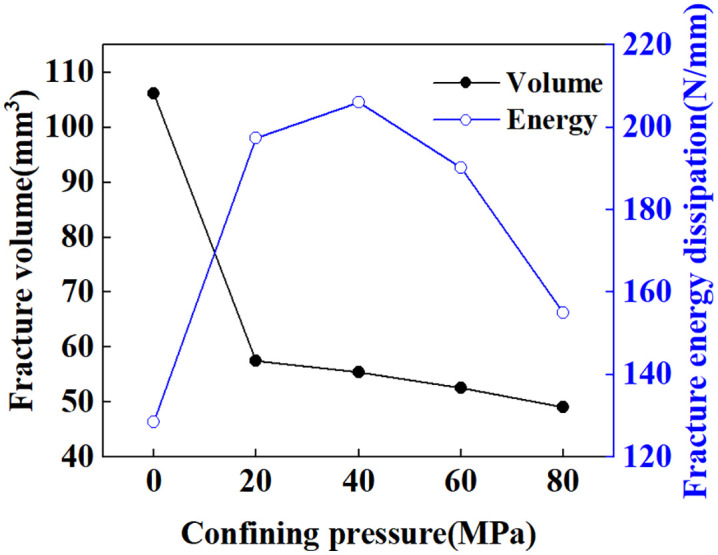
Fracture volume and fracture energy dissipation under different confining pressures.

**Fig 26:**
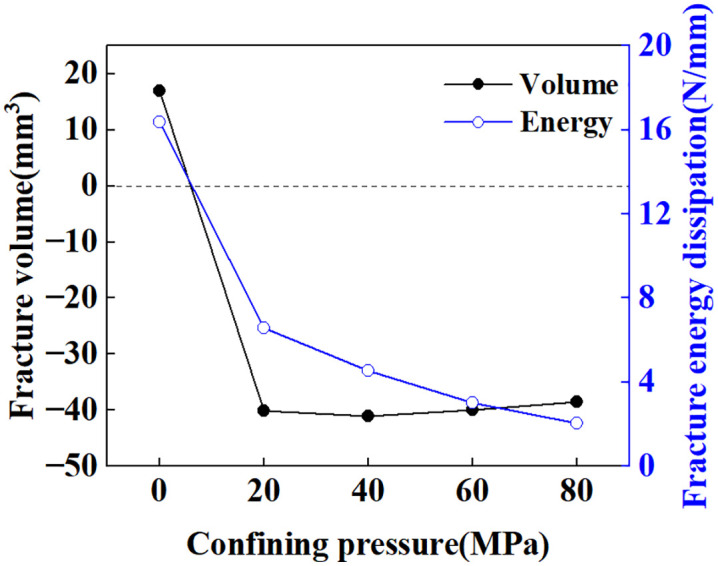
Percentage change in fracture volume and fracture energy dissipation at unloading completion relative to loading completion under different confining pressures.

**Fig 27:**
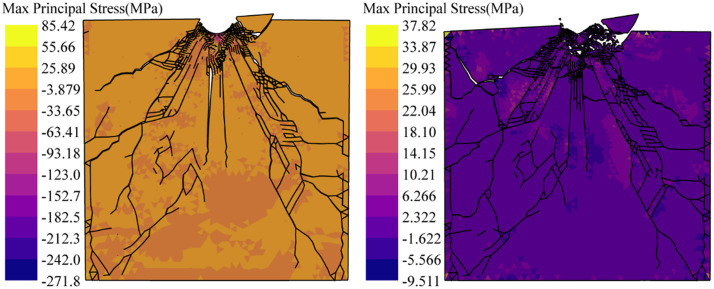
Comparison of maximum principal stress distribution at loading end and unloading end under unconfined pressure.

Fig 24 shows the crack distribution under confining pressures of 20, 40, 60, and 80 MPa, clearly demonstrating the suppression effect of confining pressure on crack propagation. Statistical results for fracture volume and fracture energy dissipation at the completion of loading (t = 10s) under different confining pressures are shown in Fig 25. The confining pressure significantly inhibits the increase in crack volume. At 20 MPa, the crack volume decreased by 45.77% compared to the case without confining pressure. However, as confining pressure further increases, the crack volume change becomes negligible. At 80 MPa confining pressure, the reduction in crack volume compared to 20 MPa is only 14.70%. From the perspective of energy dissipated during fracture propagation, due to the competing relationship between crack growth and confining pressure constraints, crack energy dissipation first increases and then decreases, reaching its maximum value at 40 MPa. Comparison of fracture volume and fracture energy dissipation between loading completion (t = 10s) and unloading completion (t = 11s), as shown in Fig 26. During the unloading phase under unconfined pressure, the fracture volume increased by 16.93% and fracture energy dissipation rose by 16.36%. This indicates further crack propagation during unloading, attributed to the release of residual stresses [70]. As shown in Fig 27, the maximum principal stress of the specimen at the completion of unloading was significantly lower than that at the completion of loading. The percentage change in fracture volume and fracture energy under confining pressure is negative, indicating that cracks close under confining pressure during the unloading phase. This occurs because the release of residual stress and the elastic rebound of the specimen are insufficient to sustain crack propagation.

## Discussion

### The mechanism of stiffness reduction is the transformation of the failure surface

Numerical simulation results of spherical indenter indentations validated the authors’ assumption: when confining pressure exceeds the brittle-ductile transition point, the indentation stiffness of the spherical indenter decreases. The decrease is attributed to the transition of the failure surface reached by the stress path on the p-q meridian plane from the shear failure surface to the ductile failure surface as the confining pressure increases. The position of the failure plane on the p-q meridian plane largely determines the corresponding indentation stiffness under different confining pressures.

### Effect of triaxial confining pressure (compared with lateral confining pressure)

Due to stress equilibrium during the pressure preloading stage under triaxial confining pressure (oil and gas drilling conditions), the mechanical response of indentations differs significantly from that under lateral confining pressure (tunnel excavation conditions). During the penetration of the indenter, the peak force did not decrease with increasing confining pressure as observed under lateral confining conditions due to the influence of hydrostatic pressure, nor did rockburst occur under high confining pressure [35]. Due to the constraints of triaxial confining pressure, the length of side cracks propagating into indentations is significantly suppressed. Consequently, in ultra-deep and extra-deep oil and gas drilling operations, the efficiency of rock breaking using the indentation method may be low because sample surface spalling is difficult to achieve. This may render roller cone bits, which break rock through impact and crushing, unsuitable for operations in formations with high confining pressures. Because the working principle of roller cone bits is consistent with the indenter penetration discussed in this paper.

### Effect of formation deviatoric stress

In this paper, the default triaxial confining pressure values are assumed to be equal, meaning no deviatoric stress is generated during the pressure preloading stage. However, in actual formations, in-situ stress and drilling fluid column pressure are not always equal. Horizontal in-situ stress always deviates to one side, and drilling fluid column pressure may sometimes fall below the formation rock’s pore pressure (underbalanced drilling). Therefore, this study still has certain limitations in practical engineering applications. However, this does not diminish its insights into the relationship between the mechanical response of rock indentation under high confining pressure and the brittle-ductile transition behavior. In subsequent research, the influence of deviatoric stress in prestressing fields may be one of the author’s research directions.

### Engineering significance of this study

The primary focus of this study is the interaction between drill bit cutters and rock during drilling in ultra-deep formations. The objective is to reveal the triaxial mechanical behavior of rock under high confining pressure and how this behavior affects the indentation mechanical response. Furthermore, the study aims to identify the differences in mechanical response between high and low confining pressures.

A key finding is that indentation stiffness exhibits a counterintuitive decrease after the confining pressure surpasses the brittle–ductile transition point. This is an important discovery because the formation pressure in ultra-deep strata exceeds the brittle–ductile transition point for some rocks, particularly high-porosity rocks, which affects the prediction of indentation force and consequently the calculation of overall bit loading. Accurate calculation of bit loading is crucial for preventing bit back-rotation and controlling vibration. In fact, a review of oil and gas exploration in ultra-deep formations reveals that drilling through high-porosity, high-permeability clastic rocks is common, especially in deepwater drilling in the Gulf of Mexico. The triaxial compression tests in this study reached confining pressures up to 190 MPa, which adequately simulate the formation pressure environment of 10 km deep wells. Therefore, this study offers valuable insights for efficient drilling operations in deepwater and ultra-deep formations and the computation of drill bit loading. Additionally, it offers reference value for other engineering applications involving biaxial confining pressure under high confining pressure, such as mining and tunnel construction.

## Conclusions

In this paper, the mechanical response and brittle-ductile transition behavior of high-porosity rocks under ultra-high pressure were analyzed via true triaxial compression tests(TTCT) and conventional triaxial compression tests(CTCT). A high-confining-pressure spherical indentation test apparatus was developed to investigate the indentation mechanical response under high confining pressure, and the correlation between the brittle-ductile transition behavior and the indentation mechanical response was explored through numerical simulation. The results of this study indicate that:

(1) The results of CTCT indicate that as the confining pressure increases, the samples undergo three failure modes: shear failure, dilatant failure, and compactive cataclastic flow, with no distinct boundaries between them. Shear-enhanced compaction dominated by deviatoric stress can act as a transient precursor to dilatancy.
(2) The results of the spherical indenter indentation tests indicate that the indentation stiffness does not always increase with increasing confining pressure; instead, a significant decrease in stiffness occurs after the brittle-ductile transition point.
(3) Peak stress points and their corresponding strains exhibit two distinctly different failure surfaces on the p-q meridian plane, and the spatial positions of these failure surfaces on the p-q meridian plane govern the indentation stiffness response to a great extent.
(4) Compared with the DP model, the MDPC model exhibits higher calculation accuracy in the numerical simulation of indentation under high confining pressure due to the presence of the cap surface, because the DP criterion cannot account for the highly constrained compressive stress state.
(5) The release of residual stress leads to significant side crack propagation during the unloading phase of indentation tests, but this propagation is significantly inhibited under confining pressure.

## Supporting information

S1 File Data-PONE-D-26-15163R1. (RAR)
